# 培美曲塞联合铂类对比吉西他滨联合铂类治疗晚期非小细胞肺癌的*meta*分析

**DOI:** 10.3779/j.issn.1009-3419.2011.01.09

**Published:** 2011-01-20

**Authors:** 金 姜, 伦 李, 晓晶 王, 金徽 田, 权 王, 乔 林

**Affiliations:** 1 730000 兰州，兰州大学循证医学中心，兰州大学基础医学院 Evidence Based Medicine Center of Lanzhou University, School of Basic Medical Science of Lanzhou University, Lanzhou 730000, China; 2 730000 兰州，兰州大学第一临床医学院 The First Clinical Medical College of Lanzhou University, Lanzhou 730000, China; 3 730000 兰州，兰州大学第二临床医学院 The Second Clinical Medical College of Lanzhou University, Lanzhou 730000, China

**Keywords:** 培美曲塞, 吉西他滨, 铂类, 肺肿瘤, Pemetrexed, Gemcitabine, Platinum, Lung neoplasms

## Abstract

**背景与目的:**

培美曲塞联合铂类方案（PP方案）作为晚期非小细胞肺癌（non-small cell lung cancer, NSCLC）一线化疗方案的疗效是否优于吉西他滨联合铂类方案（GP方案），目前尚无定论。本研究旨在评价采用PP方案与GP方案治疗晚期NSCLC的疗效及安全性。

**方法:**

计算机检索Pubmed、EMBASE、Cochrane Library、中国期刊全文数据库、中国生物医学文献数据库、中文科技期刊全文数据库等，同时追查纳入文献的参考文献，纳入PP方案对比GP方案治疗晚期NSCLC的随机对照试验（randomized controlled trial, RCT）。根据Cochrane Handbook 5.0的质量评价标准，用RevMan 5.0软件进行统计学分析。

**结果:**

共纳入4项RCT，2, 235例患者，*meta*分析结果显示采用PP方案与GP方案治疗后在1年生存率（OR=1.09, 95%CI: 0.91-1.29）、有效率（OR=1.00, 95%CI: 0.40-2.52）等方面的差异无统计学意义，而在总生存时间（MD=0.26, 95%CI: 0.21-0.30）、脱发（OR=0.51, 95%CI: 0.39-0.66）及血液毒性等方面的差异有统计学意义。

**结论:**

PP方案与GP方案在1年生存率、有效率方面疗效相当，在总生存时间、不良反应等方面有差异，PP方案可能对不耐受血液毒性、脱发等患者更适合。

## Abstract (en)

**Background and objective:**

Whether pemetrexed plus platinum (PP) regimen is superior to gemcitabine plus platinum (GP) regimen for patients with advanced non-small cell lung cancer (NSCLC) is unclear. The aim of this study is to evaluate the efcacy and safety of PP versus GP regimens for patients with NSCLC.

**Methods:**

We searched relevant randomized controlled trials (RCTs) from Pubmed, EMBASE, Cochrane Library, Chinese Journal Full-text Database, Chinese Biomedical Literature Database, Chinese Scientifc Journals Full-text Database, and traced the related references to obtain the information that has not been found. We made quality assessment of qualifed RCTs assessed by the exclusion and inclusion criteria and used RevMan 5.0 provided by the Cochrane Collaboration to perform *meta*-analysis.

**Results:**

Four RCTs involving 2, 235 patients were identifed. Tere were no statistical differences between PP and GP regimens in one-year survival rate (OR=1.09, 95%CI: 0.91-1.29), the efciency of disease (OR=1.00, 95%CI: 0.40-2.52), but overall survival (MD=0.26, 95%CI: 0.21-0.30), alopecia (OR=0.51, 95%CI: 0.39-0.66) and hematologic toxicity were signifcantly different.

**Conclusion:**

The clinical efciency of PP and GP regimens for advanced NSCLC was similar, but the side effects were different. The toxicity of PP regimen has the tendency to be more tolerable.

近年来，中国肺癌的死亡率上升速度居高不下，已经成为我国恶性肿瘤死亡的首位原因^[1]^。据卫生部全国肿瘤防治研究办公室提供的资料，自2000年-2005年间，中国肺癌患者人数估计增加了12万人，其中，男性从2000年的26万人增加到2005年的33万人，同期女性从12万人增加到17万人。目前我国肺癌发病率每年增长26.9%，如不采取有效控制措施，预计到2025年，我国肺癌患者将达到100万，成为世界第一肺癌大国^[1]^。非小细胞肺癌（non-small cell lung cancer, NSCLC）占所有肺癌患者的75%-80%，且多数确诊时已为晚期^[2]^。目前，铂类药物联合第三代化疗药物——吉西他滨已成为晚期NSCLC患者的标准一线治疗方案^[3]^，但是吉西他滨联合铂类方案（GP方案）治疗NSCLC在总有效率、1年生存时间等方面仍然不尽如人意，而且其不良反应如血小板减少、贫血较多，影响了患者的依从性^[4]^。培美曲塞是一种新型抗代谢类抗肿瘤药物，2008年9月30日美国食品与药品监督管理局批准培美曲塞作为局部晚期或转移性NSCLC的一线治疗药物^[5]^，主要通过抑制叶酸代谢途径中多个关键酶的活性，从而影响嘌呤和胸腺嘧啶核苷的生物合成，进而影响肿瘤细胞DNA合成，达到抑制肿瘤细胞增殖的目的^[6]^。

培美曲塞联合铂类方案（PP方案）作为新的化疗方案，与一线的化疗方案GP方案相比是否有更好的疗效与安全性目前尚无定论。本研究旨在运用Cochrane系统评价的方法比较两方案治疗晚期NSCLC的有效性与安全性，以期为临床实践提供决策依据。

## 1 资料与方法

### 1.1 纳入与排除标准

#### 1.1.1 研究类型

随机对照试验（randomized controlled trial, RCT），无论是否采用盲法。

#### 1.1.2 研究对象

经病理组织学或细胞学确诊的Ⅲ期、Ⅳ期的NSCLC患者，且卡氏评分 > 60分或者体能状态（performance status, PS）评分≤2分，心、肝、肾功能基本正常。

#### 1.1.3 干预措施

培美曲塞联合铂类（顺铂或卡铂）*vs*吉西他滨联合铂类（顺铂或卡铂）。

#### 1.1.4 结局测量指标

有效率、总生存时间、1年生存率、血液学毒性、消化道毒性、脱发等。

### 1.2 检索策略

用（pemetrexed OR alimta）AND（gemcitabine OR Garza）AND（carboplatin OR cisplatin OR oxaliplatin OR nedaplatin OR le platinum）AND（lung cancer OR lung neoplasms）检索Pubmed（1966.1-2010.8）、EMBASE（1974.1-2010.8）、Cochrane Library（截至2010年第8期）；用（培美曲塞或者力比泰）并且（吉西他滨或者健择）并且（卡铂或者顺铂或者奥沙利铂或者奈达铂或者乐铂）并且（肺癌或者肺肿瘤）检索中国期刊全文数据库（CNKI, 1994.1-2010.8）、中国生物医学文献数据库（CBM, 1978.1-2010.8）、中文科技期刊全文数据库（VIP, 1989.1-2010.8），RCT检索策略遵循Cochrane系统评价手册5.0，所有检索策略通过多次预检索后确定。

### 1.3 文献筛选和资料提取

2位研究者交叉核对纳入研究的结果，对有分歧的意见通过讨论或由第3位研究者决定是否纳入。缺乏的资料通过电话或信件与作者联系予以补充。提取的信息资料主要包括：①一般资料：题目、作者姓名、发表日期、文献来源和参加中心数；②研究特征：研究对象的一般情况、各组患者的基线可比性及干预措施；③结局指标。

### 1.4 质量评价

按照Cochrane评价手册5.0评价RCT质量的评价标准^[7]^，2位评价员独立对6条质量评价标准进行评价：①随机分配方法；②分配方案隐藏；③对研究对象、治疗方案实施者和研究结果测量者采用盲法；④结果数据的完整性；⑤选择性报告研究结果；⑥其它偏倚来源。针对每一项研究结果，对上述6条内容做出“是”（低度偏倚）、“否”（高度偏倚）和“不清楚”（缺乏相关信息或偏倚情况不确定）的判断。完全满足上述6条质量标准，即为“正确或充分”者，其发生各种偏倚的可能性最小，质量为A级；≥上述1条描述不清楚者为部分满足，质量为B级；≥上述1条未描述者有发生相应偏倚的可能性，质量为C级。

### 1.5 统计分析

采用国际Cochrane协作组提供的Revman 5.0软件，计数资料采用优势比（odds ratio, OR）为疗效分析统计量；计量资料采用均数差（mean difference, MD）。各效应量均以95%可信区间（confidence interval, CI）表示。各纳入研究结果间的异质性采用*Chi*^*2*^检验。当各研究间无统计学异质性（*P* > 0.1, *I*^*2*^ < 50%），采用固定效应模型对各研究进行*meta*分析；如各研究间存在统计学异质性（*P* < 0.1, *I*^*2*^ > 50%），分析其异质性来源，对可能导致异质性的因素进行亚组分析，若两项研究组之间存在统计学异质性而无临床异质性或差异无统计学意义时，采用随机效应模型进行分析。异质性源于低质量研究，进行敏感性分析。如两组间异质性过大或无法找寻数据来源时，采用描述性分析。

## 2 结果

### 2.1 检索结果

根据制定的检索式初检到相关文献143篇，通过阅读文题和摘要，排除不符合纳入标准的研究68篇，初步纳入相关研究75篇；通过阅读全文，排除非RCT 45篇及不符合纳入标准的RCT研究26篇，最终纳入RCT研究4篇^[8-11]^。文献的筛选过程见图 1。4项RCT研究共纳入患者2, 235例，进入分析的研究两组基线资料均具有可比性，一般情况见表 1。

**1:**
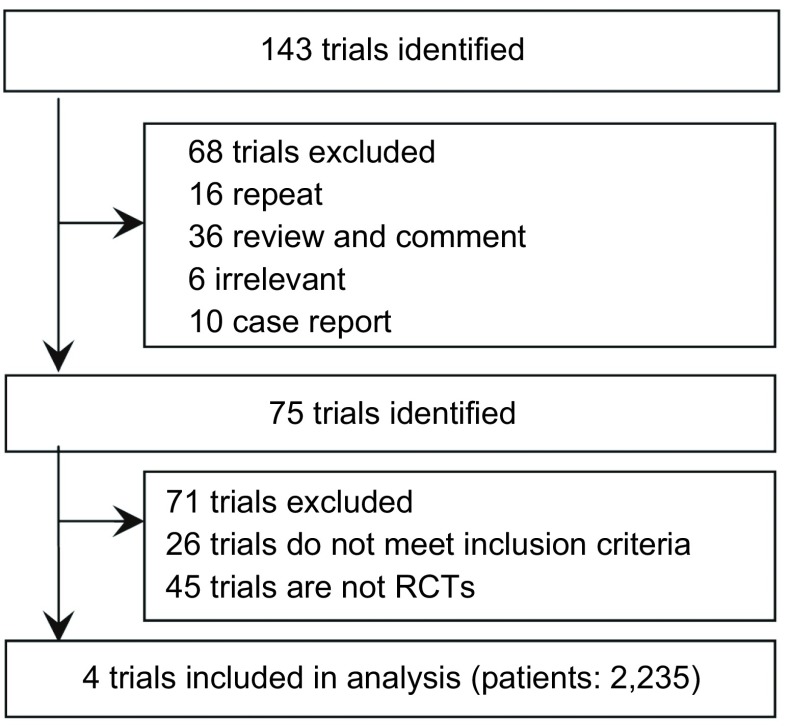
纳入研究流程图 Selection of trials

**1:** 纳入研究的一般特征 The characteristics of included studies

Included studies	Cases		Male/Female (*n*/*n*)	Age (years)			Intervations (dose)			Stages of lung cancer	
	PP	GP		PP	GP		PP	GP		Ⅲ	Ⅳ
Zheng W 2008^[8]^	23	22	14/15	67.5±5.9			Pemetrexed (500 mg/m^2^) +carboplatin (AUC: 5) for up to two cycles	Gemcitabine (1, 000 mg/m^2^)+carboplatin (AUC: 5) for up to two cycles		45	/
Wu W 2010^[9]^	14	15	18/27	55	59		Pemetrexed (500 mg/m^2^) +cisplatin (75 mg/m^2^) for up to two cycles	Gemcitabine (1, 000 mg/m^2^)+cisplatin (75 mg/m^2^) for up to two cycles		16	13
Gronberg BH 2009^[10]^	219	217	185/251	64	66		Pemetrexed (500 mg/m^2^)+carboplatin (AUC: 5) for up to four cycles	Gemcitabine (1, 000 mg/m^2^)+carboplatin (AUC: 5) for up to four cycles		124	312
Scagliotti GV 2008^[11]^	862	863	515/1, 210	61	61		Pemetrexed (500 mg/m^2^)+cisplatin (75 mg/m^2^) for up to two cycles	Gemcitabine (1, 000 mg/m^2^)+cisplatin (75 mg/m^2^) for up to two cycles		415	1, 310
AUC: area under the curve (Calvert's formula); PP: pemetrexed plus platinum; GP: gemcitabine plus platinum.											

### 2.2 纳入研究的质量评价

纳入的研究均为RCT，其中1项研究^[8]^采用随机数字表法，1项研究^[11]^采用动态随机化法，1项研究^[10]^为区组随机；纳入研究均未描述是否对分配方案进行隐藏、是否实施盲法和是否有其它可能的偏倚；2项研究^[10, 11]^报道了数据缺失，未进行意向性治疗（intention-to-treat analysis, ITT）分析；纳入研究均无选择性报告结果（图 2）。3项研究^[8, 10, 11]^被评为B级，1项研究^[9]^被评为C级。

**2:**
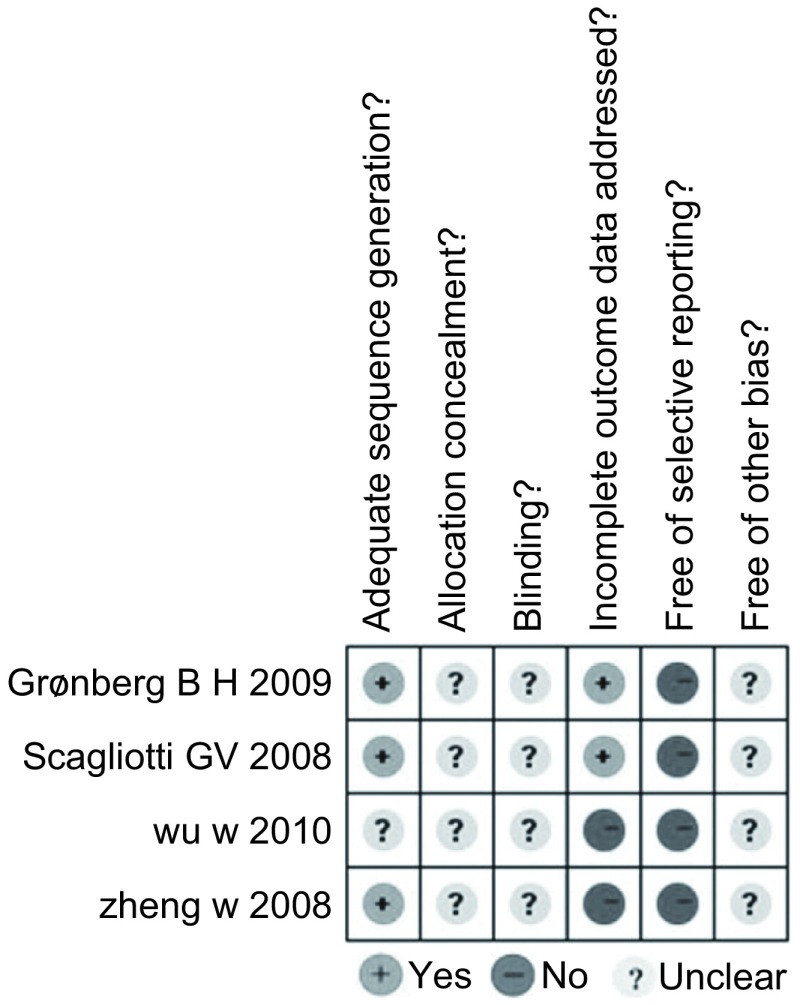
纳入研究的质量评价 Quality evaluation of included trials

### 2.3 *meta*分析

#### 2.3.1 1年生存率（图 3）

**3:**
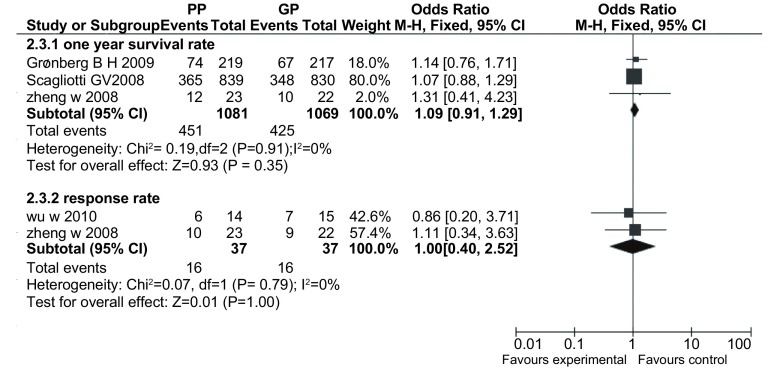
PP方案与GP方案治疗晚期NSCLC的1年生存率、有效率的情况 The one-year survival rate, response rate with pemetrexed plus platinum *vs* gemcitabine plus platinum for advanced non-small cell lung cancer (NSCLC)

3项研究^[8, 10, 11]^报道了1年生存率，各研究间无统计学异质性（*P*=0.91, *I*^*2*^=0%），采用固定效应模型，*meta*分析结果显示两方案在1年生存率方面的差异无统计学意义（OR=1.09, 95%CI: 0.91-1.29）。

#### 2.3.2 有效率（图 3）

2项研究^[8, 9]^报道了疾病治愈有效率，各研究间无统计学异质性(*P*=0.79, *I*^*2*^=0%），采用固定效应模型，*meta*分析结果显示两方案在疾病有效率方面的差异无统计学意义（OR=1.00, 95%CI: 0.40-2.52）。

#### 2.3.3 总生存时间（图 4）

**4:**
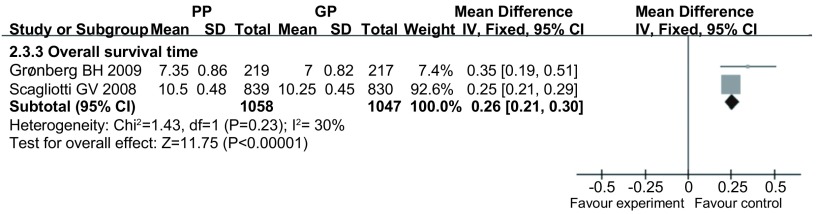
PP方案与GP方案治疗晚期NSCLC的总生存时间 The overall survival time with pemetrexed plus platinum *vs* gemcitabine plus platinum for advanced NSCLC

2项研究^[10, 11]^报道了总生存时间，各研究间无统计学异质性（*P*=0.23, *I*^*2*^=30%），采用固定效应模型，*meta*分析结果显示PP方案的总生存时间比GP方案长（MD=0.26, 95%CI: 0.21-0.3）。

#### 2.3.4 粒细胞减少（图 5）

**5:**
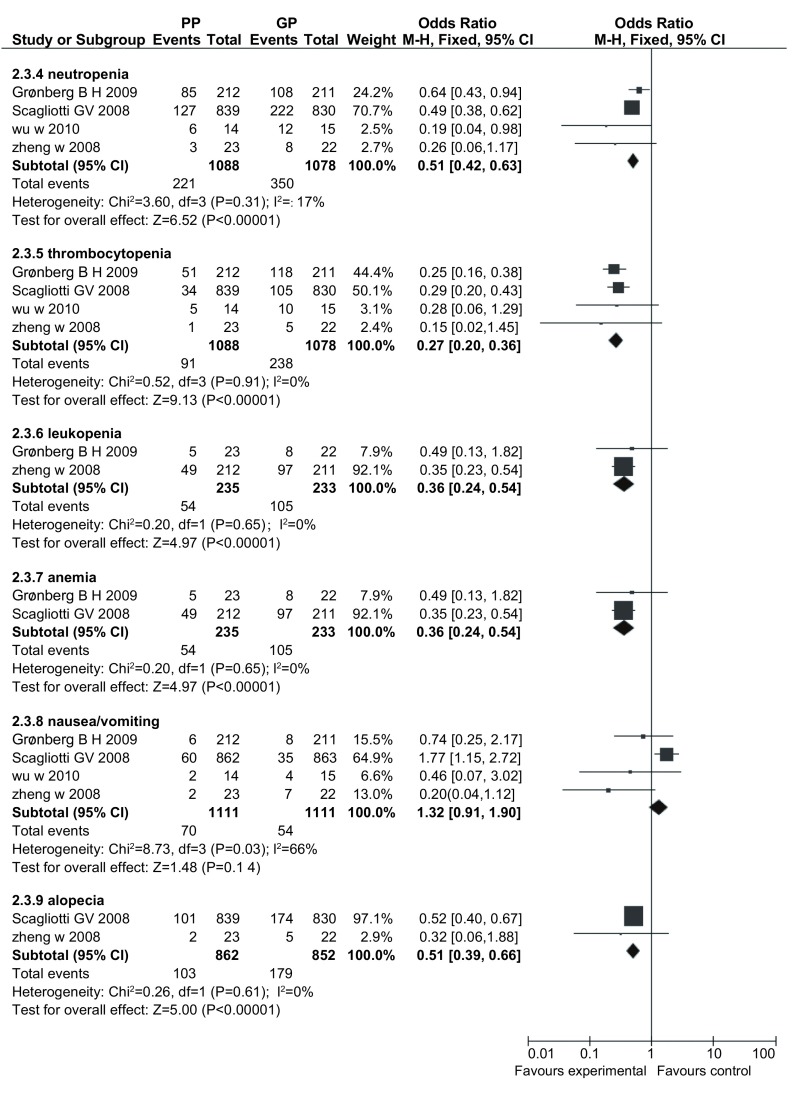
PP方案与GP方案治疗晚期NSCLC粒细胞减少、血小板减少、白细胞减少、贫血、恶心呕吐、脱发的发生情况 The neutropenia, thrombocytopenia, leukopenia, anemia, nausea/vomiting, alopecia with pemetrexed plus platinum *vs* gemcitabine plus platinum for advanced NSCLC

4项研究^[8-11]^报道了粒细胞减少，各研究间无统计学异质性（*P*=0.31, *I*^*2*^=17%），采用固定效应模型，*meta*分析结果显示PP方案导致的粒细胞减少明显少于GP方案（OR=0.51, 95%CI: 0.42-0.63）。

#### 2.3.5 血小板减少（图 5）

4项研究^[8-11]^报道了血小板减少，各研究间无统计学异质性（*P*=0.43, *I*^*2*^=0%），采用固定效应模型，*meta*分析结果显示PP方案导致的血小板减少明显少于GP方案（OR=0.38, 95%CI: 0.31-0.47）。

#### 2.3.6 白细胞减少（图 5）

2项研究^[8, 10]^报道了白细胞减少，各研究间无统计学异质性（*P*=0.65, *I*^*2*^=0%），采用固定效应模型，*meta*分析结果显示PP方案导致的白细胞减少明显少于GP方案（OR=0.36, 95%CI: 0.24-0.54）。

#### 2.3.7 贫血（图 5）

2项研究^[10, 11]^报道了贫血，各研究间有统计学异质性（*P*=0.06, *I*^*2*^=71%），采用随机效应模型，*meta*分析结果显示两方案在贫血的差异无统计学意义（OR=0.72, 95%CI: 0.38-1.35）。

#### 2.3.8 恶心呕吐（图 5）

4项研究^[8-11]^报道了恶心呕吐，各研究间有统计学异质性（*P*=0.03, *I*^*2*^=61%）。采用随机效应模型，*meta*分析结果显示两方案在恶心呕吐方面差异无统计学意义（OR=1.32, 95%CI: 0.91-1.90）。

#### 2.3.9 脱发（图 5）

2项研究^[8, 11]^报道了脱发，各研究间无统计学异质性（*P*=0.61, *I*^*2*^=0%），采用固定效应模型，*meta*分析结果显示PP方案导致的脱发少于GP方案（OR=0.51, 95%CI: 0.39-0.66）。

## 3 讨论

*meta*结果显示：在疗效方面，与GP方案相比，PP方案在治疗晚期NSCLC的有效率和1年生存率方面的差异无统计学无意义，但延长了总生存时间；在不良反应方面，PP方案的血小板减少、白细胞减少、粒细胞减少、贫血和脱发等事件发生率较GP方案低，而在消化道毒性如恶心呕吐方面，两方案的差异无统计学意义。

20世纪90年代以来，第三代化疗药物如GP方案已成为晚期NSCLC的标准一线治疗方案^[12]^。吉西他滨是嘧啶类抗代谢药物，研究^[13, 14]^表明其对多种实体瘤如NSCLC疗效较好。但是GP方案具有相对高的致白细胞和血小板减少的骨髓毒性，影响了临床的推广使用。随着新型多靶点抗叶酸药物培美曲塞的出现，培美曲塞联合铂类的新的化疗方案是否具有更好的疗效和安全性，成为临床医师关注的课题。

培美曲塞是一种新型的多靶点抗叶酸药物，作用于叶酸依赖性代谢途径中的多个酶，包括胸苷酸合成酶，二氢叶酸还原酶和甘氨酰胺核苷甲酰基转移酶等，导致肿瘤细胞的嘌呤和嘧啶合成受阻，最终使肿瘤细胞的增殖停滞于S期，因而具有抗癌作用^[15]^。而且，培美曲塞还具有显著的抗叶酸代谢能力，该作用强度比其它的抗叶酸药物如氨甲喋呤高100倍^[16]^，所以PP方案在总生存时间方面长于GP方案，一方面是由于以上原因，另一方面Grø nberg等^[10]^的研究中采用PP方案的肺癌患者PS=2的患者的比例（22%）少于采用PP方案的PS=2的患者，因为有研究^[17]^揭示PS=2的患者是一个明显的影响预后的危险因素。同时由于培美曲塞抑制叶酸依赖酶，故可能产生严重的不良反应，Vogelzang等^[18]^的研究提示培美曲塞联合铂类在用药前补充叶酸和维生素B_12_，培美曲塞组的骨髓毒性和胃肠道毒性显著减少。郑伟^[8]^报道显示，PP方案在血液学毒性、消化道毒性等方面的毒性反应减少方面有一定的优势。

纳入的4项研究^[8-11]^全部都是RCT，同质性较好。纳入研究均提及随机分配，有3项研究^[8, 10, 11]^描述具体随机方法。纳入研究均未提及分配隐藏。纳入研究也均未描述盲法和失访，这将产生测量和实施偏倚。4项研究中培美曲塞的剂量相同，但是吉西他滨的剂量不同，可能对结果有影响。4项研究总纳入的患者相对较少，患者分别来自中国、美国、挪威，尚需要其它国家和地区类似的高质量的多中心RCT进一步证实。

建议今后临床研究应该：①试验前进行严格的试验设计，减少偏倚；②采用客观、国际认可的且有利于交流的终点疗效指标，并按照CONSORT标准报告试验。

当前研究显示，PP方案治疗晚期NSCLC对比当今的一线化疗方案在疗效方面相似，但是可减少一些毒副反应如血小板减少、贫血等，我们倾向于认为PP方案与GP方案相比，疗效相似，但是耐受性更好，对于身体状况不佳的老年患者以及不能耐受贫血、脱发、血小板减少的患者，PP方案可能更适合作为治疗晚期NSCLC的一线化疗方案。
